# Low-burden speech-based screening of depressive symptoms using ultra-short speech samples

**DOI:** 10.3389/fpsyt.2026.1888425

**Published:** 2026-09-23

**Authors:** Zhiguo Zheng, Lijuan Liang, Jin Zhang, Kai Qi, Guanjun Wang, Chenyang Xue

**Affiliations:** 1 School of Information and Communication Engineering, Hainan University, Haikou, China; 2 School of Electronic Science and Technology, Hainan University, Haikou, China; 3 Department of Psychology, The First Affiliated Hospital of Hainan Medical University, Haikou, China; 4 School of Information Engineering, Hainan Vocational University of Science and Technology, Haikou, China; 5 School of Intelligent Medicine and Technology (Big Data Research Center), Hainan Medical University, Haikou, China

**Keywords:** depressive symptom screening, feature-model matching, low-burden screening, positive recall task, speech analysis, time-window optimization

## Abstract

**Background:**

Speech holds promise for depressive symptom screening, but practical applications require clearer guidance on effective segmentation, optimal speech tasks, minimum duration, and feature-model matching.

**Objective:**

To enable low-burden screening for depressive symptoms, this study aims to evaluate the effectiveness of short speech samples and systematically investigate how to identify the optimal practical combination of different speech tasks, time-window lengths, and the matching between features and models.

**Methods:**

Using the HMU-DDAC-24 dataset of 143 participants aged 18–22 years, we evaluated 10 segmentation lengths (0.1–15 s), 5 models (Random Forest, Extreme Gradient Boosting (XGBoost), Long Short-Term Memory (LSTM), Transformer, Extended Long Short-Term Memory (xLSTM)), and 3 feature types (frame-level, statistical, fused). We also assessed 19 window lengths (1–35 s) across four speech tasks and their combinations. In addition, supplementary cross-dataset validation analysis was performed using the external Emotional Audio-Textual Depression (EATD) dataset.

**Results:**

Frame-level features performed best with sequence models and short windows (0.1–2 s; Transformer accuracy: 0.731 at 2 s). Statistical features paired best with tree models and short windows (Random Forest accuracy: 0.698 at 1 s). Fused features improved tree models (Random Forest accuracy: 0.703 at 4 s) but introduced noise into sequence models. Task 4 (positive recall) achieved the best overall performance (Area Under the Receiver Operating Characteristic Curve (AUC) = 0.860), outperforming all single and combined tasks, showing no multi-task synergy. A 1-s segment performed comparably to full-length speech (3.14% relative performance loss). Kullback-Leibler divergence analysis showed short segments closely approximated the full recording’s distribution (Task 4 KL = 9.56 at 1 s).

**Conclusions:**

When combined with appropriate speech tasks and feature-model matching, short speech samples can facilitate low-burden screening for depressive symptoms. Supplementary analysis on the EATD dataset supported these trends, though absolute performance depended on feature representation. We recommend positive recall with 2-s truncation, frame-level features, and Transformer for accuracy-oriented settings; and 1-s truncation, statistical features, and Random Forest for resource-limited settings. These findings may inform rapid, remote speech-based screening for depressive symptoms, especially in resource-limited primary-care settings.

## 1 Introduction

### 1.1 Background

Mental disorders affect nearly one billion people worldwide, and depression represents a major contributor to the global mental health burden (1). However, current screening and diagnostic methods still rely primarily on clinical interviews and rating scales, such as the Patient Health Questionnaire-9 (PHQ-9) and the 17-item Hamilton Depression Rating Scale (HAM-D-17), which are inherently subjective and require substantial clinical resources (2, 3). In digital mental health and remote screening settings, there is a growing need for efficient, low-burden screening methods to reduce the strain on healthcare resources and support early identification. As a non-invasive and easily obtainable biomarker that can be collected remotely, speech has attracted increasing attention in depressive symptom screening (4–6). Psychomotor retardation associated with depression may affect vocal fold tension, airflow control, and phonation dynamics, which may in turn be reflected in measurable acoustic changes in speech (7–9).

### 1.2 Research gaps and core scientific questions

Although significant progress has been made in speech-based depressive symptom screening, four key challenges remain to be addressed before it can be translated into practical digital mental health applications:

Issue 1: Speech Segmentation

Fixed-frame operations (a 25-ms Hamming window and a 10-ms frame shift) are commonly used in speech recognition tasks (10, 11). However, individuals with depressive symptoms may exhibit slow speech, flat intonation, and low vocal energy. Systematic research on targeted segmentation and on the impact of different segmentation methods on model performance is still lacking.

Issue 2: Task Selection:

Most studies employ complex combinations of cognitive and emotional tasks (12–14), but few have examined whether such multi-task combinations truly provide information gain or merely introduce redundant noise. Is a single task sufficient? Which task is optimal?

Issue 3: Speech Duration:

The conventional view holds that longer recordings provide richer statistical distributions (15–17). However, long recordings can easily lead to participant fatigue and contain a large number of silent segments and macrosyntactic noise. There is a lack of quantitative evidence regarding the minimum speech duration required for accurate detection.

Issue 4: Feature-Model Matching:

Many studies simply concatenate frame-level dynamic features with global statistical features (18, 19), yet overlook the issue of inductive bias matching between feature types and model architectures. Unlike previous studies that primarily benchmark accuracy on specific datasets, the present work systematically compares tasks, temporal windows, and feature-model combinations to provide actionable design rules that can guide the development of screening tools across diverse application contexts.

To address these issues, this study aims to systematically investigate:

1. the impact of different speech segmentation methods on model performance;
2. which speech tasks most effectively elicit depressive symptom-related acoustic features and whether multi-task combinations yield synergistic gains;
3. the minimum speech duration required for accurate detection; and
4. the optimal matching patterns between different feature types and model architectures.

### 1.3 Main contributions

To address the aforementioned issues, this study utilized the HMU-DDAC-24 dataset to systematically evaluate the impact of different segmentation methods, task types, time windows, and feature-model combinations on the results.

Distinguishing our work from previous studies that primarily report classification accuracy on specific datasets, we frame our contribution around design principles, systematically demonstrating how task choice, window length, and feature-model matching jointly determine performance, and under what conditions ultra-short windows can be effective.

The main contributions are as follows:

1. Dataset Contribution
  We constructed the HMU-DDAC-24 dataset, a clinically collected speech corpus comprising four cognitive-emotional tasks.
2. Methodological Contribution
  We systematically reveal the coupling patterns among features, models, and windows, and propose clear matching criteria: frame-level features + sequence models + short windows (0.1–2 seconds) to achieve the performance ceiling; statistical features + tree models + medium-to-long windows (4 seconds) as a robust baseline.
3. Theoretical Contribution
  We present evidence that acoustic information associated with depressive symptoms is preserved even within very short speech windows. Further KL divergence analysis confirms that short-window speech shares the same feature distribution as longer speech, suggesting that short speech segments are sufficient to retain effective acoustic information for screening depressive symptoms.
4. Clinical Contribution
  We provide practical and deployable screening configurations: an efficiency-maximizing configuration (positive recall task + 2 s truncation + frame-level features + Transformer) and a resource-constrained configuration (positive recall + 1 s truncation + statistical features + Random Forest).

In addition, we conducted cross-dataset validation on the EATD dataset (20). The results indicate that when strictly using the 592-dimensional features from the main experiment, the results exhibit limited transferability to EATD. However, after adopting an enhanced feature representation better suited to EATD, we evaluated the same three core trends observed in the main experiment: differences across tasks, the superiority of short time windows, and the lack of multi-task synergy.

Recent work has also highlighted the importance of data-collection design and short speech snippets in voice-based depression detection (21). While their study focused on improving classification accuracy through data collection framework optimization, our work extends this direction by systematically investigating the joint optimization of task selection, temporal window length, and feature-model matching.

## 2 Materials and methods

### 2.1 Participants

The 143 participants in this study (aged 18–22) were recruited from the First Affiliated Hospital of Hainan Medical University; all participants signed informed consent forms as required. The study protocol was approved by the Ethics Committee of the First Affiliated Hospital of Hainan Medical University (Approval No.: 2024-KYL-133) and adhered to the principles of the Declaration of Helsinki. The demographic and clinical characteristics of the participants are presented in **Table 1**.

**Table 1:** Demographic and clinical characteristics of participants.

Characteristic	HC (n = 72)	Mild (n = 42)	Moderate (n = 29)	Test statistic	P value
Age (years), mean ± SD	19.87 ± 1.05	19.62 ± 1.08	19.55 ± 1.16	F(2,140) = 1.12	0.329
Sex, n (%)				χ²(2) = 0.15	0.928
Male	32 (44.4%)	19 (45.2%)	15 (51.7%)		
Female	40 (55.6%)	23 (54.8%)	14 (48.3%)		
PHQ-9 score, mean ± SD	1.94 ± 1.36	7.21 ± 1.45	12.07 ± 1.38	F(2,140) = 523.4	< 0.001

HC, healthy controls; Mild, participants with mild depressive symptoms (PHQ-9 scores of 5–9); Moderate, participants with moderate depressive symptoms (PHQ-9 scores of 10–14). Between-group comparisons were conducted using one-way analysis of variance for continuous variables, including age and PHQ-9 score, and the chi-square test for categorical variables, including sex. SD, standard deviation; PHQ-9, Patient Health Questionnaire-9.

Inclusion criteria for the depressive symptoms group: PHQ-9 total score ≥ 5 (22). This threshold is widely used to identify individuals with elevated depressive symptoms and has particularly good validity in adolescent and young adult populations. In this study, participants in this group had PHQ-9 scores ranging from 5 to 14, corresponding to mild to moderate depressive symptoms.

It should be noted that this study did not use standardized diagnostic interviews to confirm clinical diagnoses in participants; therefore, this sample should be considered a non-clinical group with depressive symptoms.

Inclusion criteria for the healthy control group: (1) No personal or family history of any mental illness; (2) PHQ-9 score < 5; (3) Other exclusion criteria were the same as those for the depressive symptoms group.

Exclusion criteria (applicable to all participants): (1) self-reported or clinically recorded history of other mental disorders; (2) Severe physical or neurological illness; (3) Substance abuse or dependence.

### 2.2 Task design

To elicit distinct cognitive and affective states, we constructed the Hainan Medical University Depression Diagnosis Audio Corpus 2024 (HMU-DDAC-24), recorded in 2024 at the Department of Medical Psychology, the First Affiliated Hospital of Hainan Medical University. The corpus included four speech tasks designed to induce different emotional states: (1) short passage reading (T1), in which participants read the neutral passage *The North Wind and the Sun* (23) to provide standardized speech samples; (2) daily recall (T2), in which participants answered open-ended questions about recent daily experiences; (3) negative recall task (T3) (24, 25), in which participants described the saddest memory from the past two weeks; and (4) positive recall task (T4) (26), in which participants described the happiest memory from the past two weeks.

The mean duration of the full recordings was 22.15 ± 16.64 seconds (range: 2.16–60.86 seconds) across all participants and tasks. By group, the HC group had a mean duration of 25.97 ± 15.38 seconds, while the Depression group had 19.82 ± 17.09 seconds. By task type, the fixed reading task was the longest (HC: 42.18 ± 6.17 s; Depression: 43.21 ± 8.56 s), followed by the positive recall task (HC: 20.22 ± 13.20 s; Depression: 11.43 ± 9.69 s), the negative recall task (HC: 19.65 ± 13.33 s; Depression: 12.51 ± 11.81 s), and the daily recall task was the shortest (HC: 12.17 ± 12.38 s; Depression: 12.13 ± 11.54 s). Notably, 87.6% of all recordings (879 out of 1,003) had durations of 35 seconds or less; extending the analysis beyond 35 seconds would therefore exclude a substantial portion of the data. This informed our choice of 35 seconds as the upper bound for the duration analysis. These statistics were derived from 1,003 recordings across 143 participants.

### 2.3 Data pre-processing feature extraction

Raw audio recordings were uniformly resampled to 16 kHz, followed by Wiener filtering using scipy.signal.wiener for noise reduction, silence removal via librosa.effects.split (top_db=20), and segmentation into non-overlapping fixed-length windows (0.1–15 s for feature-model-window evaluation; 1–35 s for duration analysis). Segments shorter than the target length were zero-padded. All preprocessing was implemented using librosa (v0.10.2) and SciPy.

In total, 216-dimensional feature vectors were extracted for each recording by concatenating 188 frame-level features (27) and 28 statistical features (28, 29). The frame-level features consisted of 128-dimensional Mel-spectrogram features (30) and 60-dimensional enhanced Mel-frequency cepstral coefficient (MFCC) features (20 base MFCCs plus their first- and second-order derivatives) (31–33). The statistical features comprised 8 F0 statistics, 6 zero-crossing rate statistics, 6 short-time energy statistics, and 8 spectral statistics. The detailed definition and dimensionality of each feature type are provided in **Supplementary Table 1**.

### 2.4 Model architectures

To systematically evaluate how model architecture interacts with feature types and temporal window lengths, we selected five representative models. Random Forest (34, 35) and XGBoost (36) were included as widely-used baseline models in speech-based classification tasks. LSTM (37) was selected to capture sequential dependencies in frame-level acoustic features. Transformer (38) was included as a state-of-the-art sequence model leveraging self-attention for long-range dependencies. Finally, xLSTM (39, 40) was incorporated as a recent extension of the LSTM family that introduces exponential gating and memory mixing, offering a balanced architecture between recurrent and attention-based paradigms. This selection ensures coverage of diverse architectural families and enables us to systematically investigate feature-model matching. This is a core theme of our study. The model parameters are listed in **Supplementary Table 2**.

### 2.5 Experimental design

To address the core research questions of this study, two systematic experiments were conducted:

1. Feature-Model-Window Cross-Evaluation

To assess model performance across various segmentation strategies and feature sets, we evaluated 150 distinct conditions. These comprised combinations of 3 feature sets (188-dimensional, 28-dimensional, and 216-dimensional), 5 models (Random Forest, XGBoost, LSTM, Transformer, and xLSTM), and 10 window sizes (0.1, 0.5, 1, 2, 3, 4, 5, 6, 10, and 15 s).

2. Multi-Task Ablation Study

To evaluate the impact of different speech tasks, we tested individual tasks (T1-T4) and all possible multi-task combinations (15 combinations in total). The detailed combination schemes are provided in **Supplementary Table 3**.

For all experiments, a speaker-independent train-test split (80/20 ratio) was applied to prevent data leakage. Each experiment was repeated five times using different random seeds, and the results are reported as the mean and standard deviation.

For all models, hyperparameters were fixed rather than optimized through grid search on our specific dataset. This design choice ensures a fair comparison across the five architectures, as the focus of this study is on the relative performance of model families and their coupling with feature types and window lengths, rather than on achieving the highest possible accuracy through per-model tuning. The complete hyperparameter settings for each model are provided in **Supplementary Table 2**.

### 2.6 Validation on the EATD dataset

To assess the robustness of our experimental results across different datasets, we conducted external validation experiments on the EATD dataset, designing the following two approaches:

1. Baseline

We used the same configuration as in the main experiment (1-second segments, Random Forest classifier, 592-dimensional acoustic features). These features were constructed by concatenating 28 statistical features with the mean, standard deviation, and maximum values of 188 frame-level features.

2. Enhanced Statistical Features

While retaining the Random Forest classifier, we designed an enhanced feature set specifically for the EATD dataset. Based on 188 frame-level acoustic descriptors, 12 statistical measures (mean, standard deviation, minimum, maximum, median, Q25, Q75, range, interquartile range, skewness, kurtosis, and coefficient of variation) were calculated for each 1-second segment, resulting in a 2,256-dimensional full statistical representation.

### 2.7 Evaluation metrics

The models were evaluated using both classification metrics (Accuracy, Precision, Recall, Specificity, F1 score, and AUC) and auxiliary analytical metrics [KL divergence (41, 42), relative loss, and synergy index (43)]. Specifically, KL divergence was employed to quantify the discrepancy in feature distributions between local short speech segments and global full-length recordings.

### 2.8 Software and hardware

All preprocessing of speech data, feature extraction, and model training were implemented using Python (v3.10.9). Demographic statistics were analyzed using SPSS 26.0 (V26.0). Deep learning models (LSTM, Transformer, and xLSTM) were trained on an NVIDIA GeForce RTX 3060 GPU (12 GB VRAM) using PyTorch (v2.3.0) with CUDA 11.8. Tree-based models (Random Forest and XGBoost) were trained on CPU using scikit-learn (v1.4.1) and XGBoost (v2.1.3), respectively. Additional Python packages used included librosa (v0.10.2) for feature extraction, numpy (v1.26.4), pandas (v2.2.3), scipy (v1.13.1) for data processing, and matplotlib (v3.9.4) with seaborn (v0.13.2) for visualization. The code has been made publicly available (see the Data availability section for details).

## 3 Results

### 3.1 Systematic comparison across features, models, and window lengths

#### 3.1.1 Frame-level features (188 dimensions): superiority of sequence models with short windows

Using only frame-level features, sequence models (Transformer, xLSTM, LSTM) achieved accuracies of 0.722–0.731, with AUC values approaching 0.80, showing a clear advantage over tree-based models(Random Forest, XGBoost), whose accuracies were 0.575–0.584 (**Supplementary Table 4**). Transformer achieved the highest recall (sensitivity) of 0.791 ± 0.063 at the 2-second window, indicating strong capability in identifying true positive cases, a critical property for screening applications where minimizing false negatives is important.The optimal window lengths for the sequence models were concentrated in the 0.1–2s range (xLSTM performed best at 0.1s, and Transformer at 2s), whereas the tree-based models relied on longer 4s windows to reach their peak performance.

#### 3.1.2 Statistical features (28 dimensions): revisiting tree-based models and “window compensation” in sequence models

After switching to statistical features, the performance of tree models improved significantly (Random Forest accuracy: 0.698; XGBoost accuracy: 0.664), while the accuracy of sequence models dropped to 0.697–0.705 (**Supplementary Table 5**).

After changing the feature dimension, the two model types exhibited the following significant changes in time windows:

1. “Window compensation” in Sequence Models.
  Upon losing frame-level dynamic information, the optimal window size for sequence models increased sharply (Transformer surged from 2 seconds to 10 seconds, xLSTM extended from 0.1 seconds to 15 seconds, and LSTM also lengthened from 1 second to 2 seconds). This reveals a “compensation mechanism” whereby sequence models, having lost their temporal advantage, are forced to accumulate differences in statistical characteristics by extending the input window.
2. “Window release” in Tree Models.
  Conversely, the optimal window for Random Forest shrinks significantly from 4 seconds at the pure frame level to 1 second. This is because the statistical features themselves already highly condense macro-level distribution information; tree models require only a very short time (1 second) to obtain sufficient classification information, eliminating the need to rely on long windows to passively accumulate data.

#### 3.1.3 Fused features (216 dimensions): “double-edged sword” effect and inherent model biases

When evaluating fused features (188-dimensional frame-level + 28-dimensional statistical), simple feature concatenation exhibited a clear “double-edged sword” effect (**Supplementary Table 6**), which was particularly evident in terms of the balance of clinical screening performance across models:

1. Tree-based models clearly benefit, with more balanced depressive symptoms screening profiles.
  Owing to the stable global anchors provided by the statistical component, Random Forest accuracy increased from 0.584 with pure frame-level features to 0.703, and XGBoost accuracy improved to 0.682. More importantly, the fused features substantially corrected the imbalance observed with tree-based models using only frame-level inputs. For example, the F1 score of Random Forest increased from 0.577 to 0.704, transforming it from a relatively underperforming classifier into a more clinically useful and balanced model.
2. Sequence models are perturbed, with degraded overall performance.
  In contrast, the overall performance of sequence models was negatively affected. The accuracy of the Transformer decreased from 0.731 to 0.709, and its previously strong overall classification performance (F1 score) dropped from 0.722 to 0.692. This suggests that repeating global static statistics across time steps acts as “feature noise” for sequence models, thereby impairing their ability to capture meaningful temporal dynamics. Model AUCs across different feature sets are shown in **Figure 1A**.
3. xLSTM demonstrates strong robustness to interference.
  Despite the general degradation among sequence models, xLSTM maintained a high accuracy of 0.722 even with an extremely short 0.1-s window. Its precision (0.742) and specificity (0.729) also remained among the highest and most balanced across all models. This further suggests that the gating mechanisms in xLSTM may confer greater robustness to redundant feature information. The AUC performance of the xLSTM model across different feature sets is shown in **Figure 1B**.
4. Window preferences remain stable.
  Despite changes in feature dimensionality and distribution, the window-length preferences of the model families remained largely unchanged: sequence models continued to favor short windows (0.1–1 s), whereas tree-based models again achieved their best performance with window lengths around 4 s.

**Figure 1:**
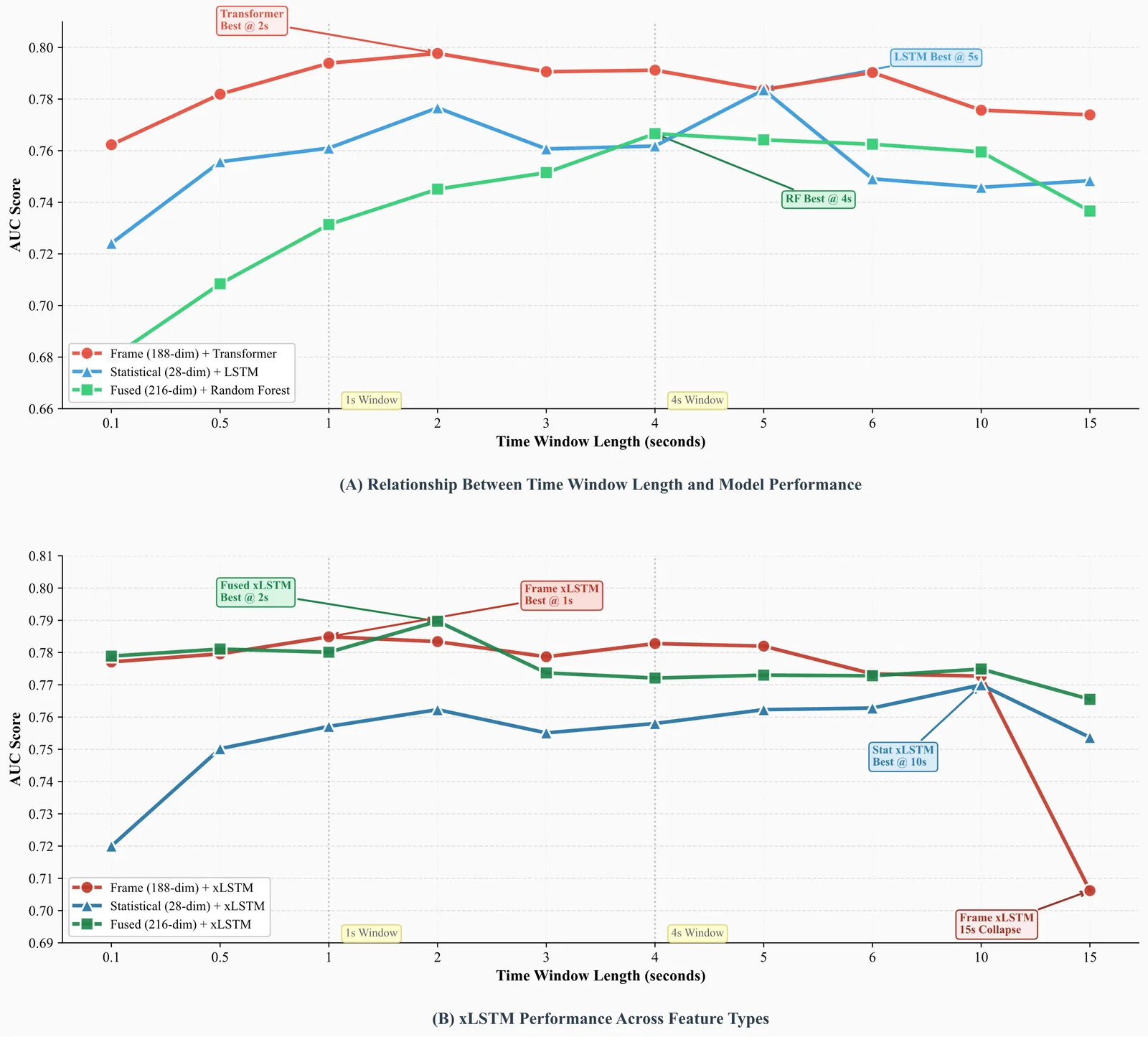
AUC performance of models across different time window lengths and feature types. **(A)** Relationship between time window length and model performance for the best-performing model under each feature type. **(B)** AUC performance of the xLSTM model across different feature types and time window lengths.

#### 3.1.4 Summary: the coupling relationship among features, models, and window length

These results reveal a coupling relationship among feature types, model architectures, and window lengths. Frame-level features are better suited to sequence models with short windows, whereas statistical features are better suited to tree-based models. Furthermore, feature fusion is a double-edged sword: it can enhance the performance of tree-based models, but it can also introduce noise into sequence models. Therefore, in depressive symptom screening, it is essential to consider the joint matching of feature type, model architecture, and window length.

### 3.2 Multi-task analysis and ablation experiments

To examine the influence of task configuration on model performance, we adopted a Random Forest classifier and expanded the feature space to 592 dimensions (mean, standard deviation, and maximum of the 188 frame-level features, plus the 28 statistical features). All single- and multi-task, as well as ablation experiments, were conducted on preprocessed 1-s speech segments.

#### 3.2.1 Single-task performance: superiority of T4

Evaluating individual tasks, T4 (positive recall) yielded the global optimum AUC of 0.860 (**Supplementary Table 7**). This indicates that depressive symptom-induced acoustic deviations are maximally discriminative during positive emotion elicitation, corroborating the clinical manifestation of anhedonia.

#### 3.2.2 Multi-task combinations: “zero synergy” effect

Evaluating the impact of multi-task integration reveals a pronounced “zero-synergy” phenomenon, where concatenating speech tasks fails to enhance classification efficacy. Benchmarked against the optimal single-task T4 (AUC = 0.860), multi-task feature fusion predominantly resulted in performance stagnation or degradation. Specifically, the leave-one-out tri-task configuration C1 (T2+T3+T4) exhibited a marginal AUC decrement to 0.859 (−0.10%), whereas the full-task baseline A (T1+T2+T3+T4) deteriorated significantly to 0.833 (−2.70%) (**Figures 2**, **3**; **Supplementary Table 8**).

**Figure 2:**
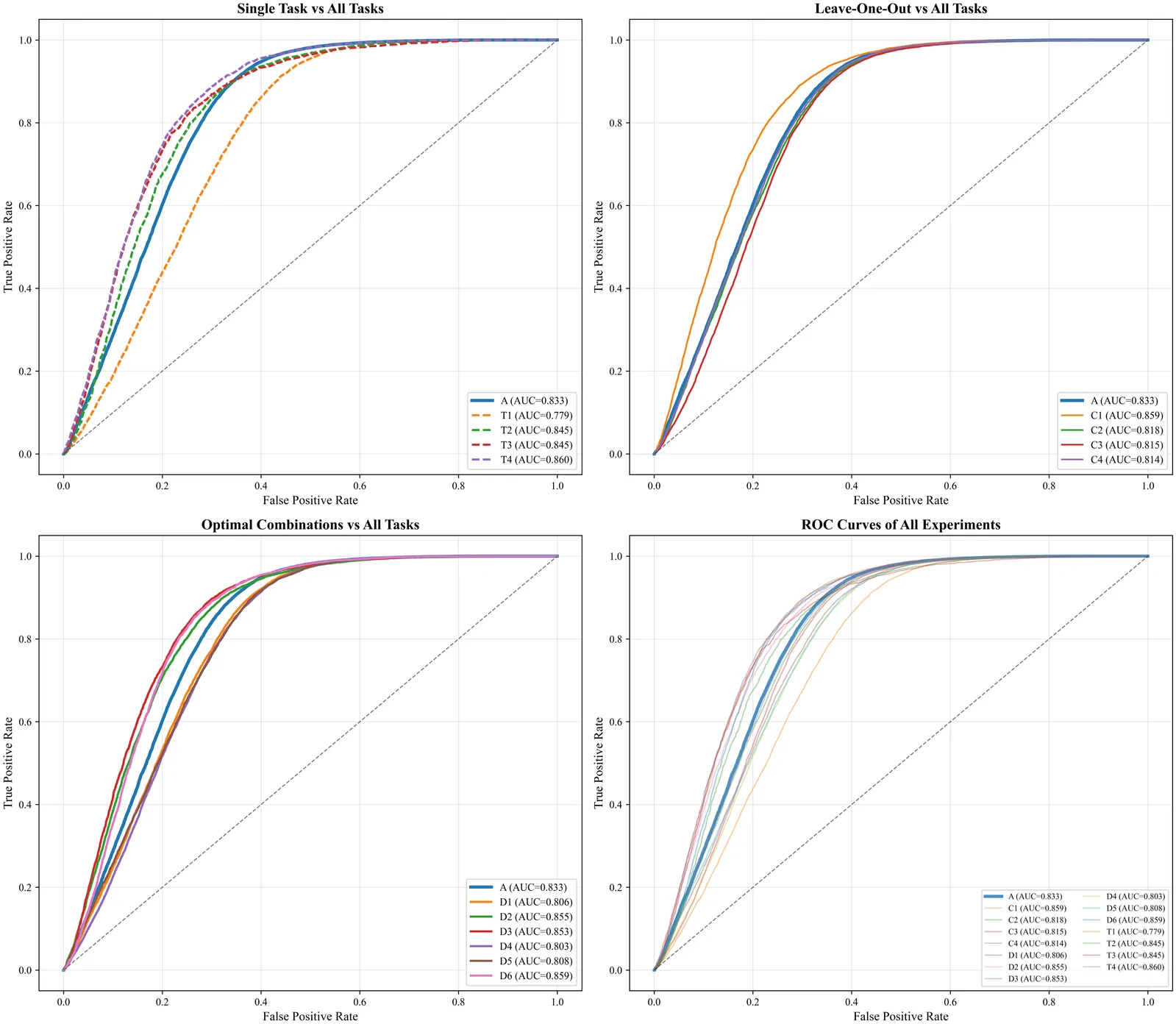
ROC curves and AUC performance across different task combinations, including single-task, leave-one-out, optimal-combination, and all-experiment comparisons.

**Figure 3:**
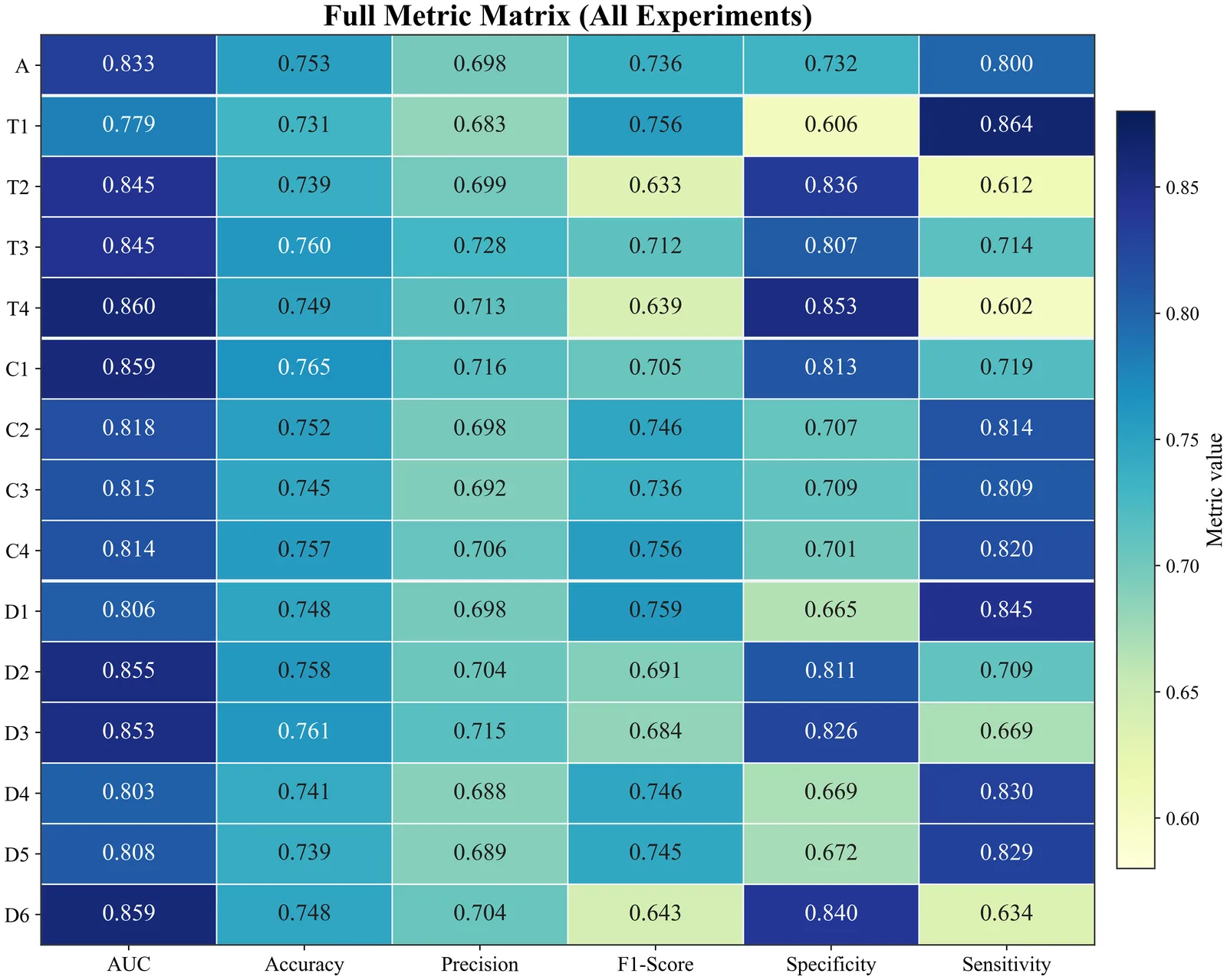
Heatmap visualization of performance metrics for all task combinations.

Quantitative synergy analysis corroborates this, with pairwise synergy indices clustering around zero (**Figure 4**). This indicates that diverse speech elicitation paradigms yield highly redundant, rather than complementary, acoustic representations of depressive symptoms. Furthermore, incorporating specific tasks (e.g., the T1 reading task) acts as noise injection, diluting discriminative affective acoustic features and causing substantial declines in both AUC and specificity. Consequently, deploying a singular, high-yield affective paradigm (e.g., T4) optimizes screening efficacy while participant cognitive load and screening duration.

**Figure 4:**
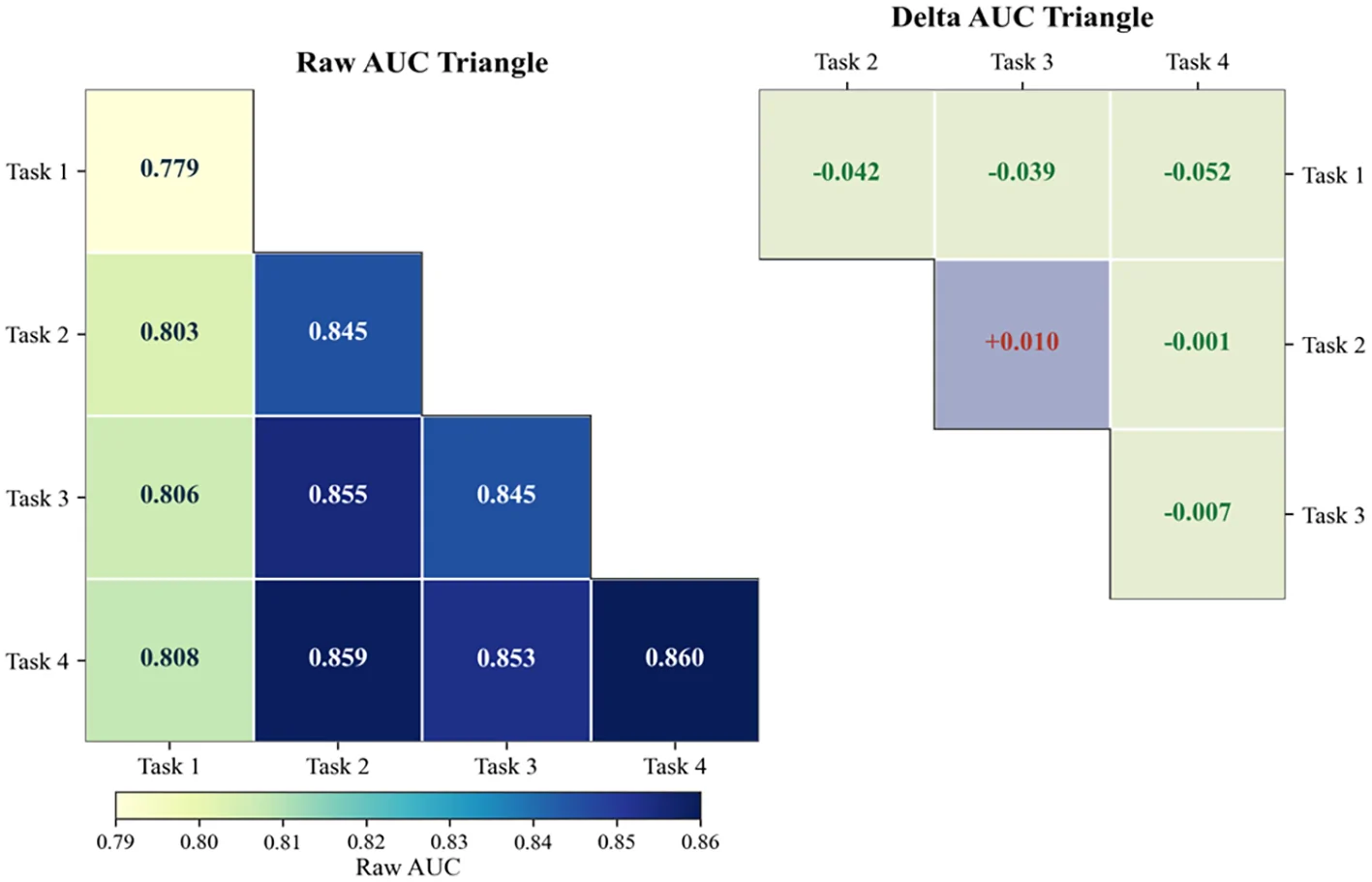
Heatmap of task synergy across different speaking tasks.

#### 3.2.3 SHAP analysis of feature importance

To further confirm that the classification model captures meaningful speech markers of depressive symptoms rather than task-specific noise, we performed a SHAP analysis using the optimal configuration (T4, 1-second window, Random Forest) and extended it to all four tasks (T1–T4) to assess cross-task consistency.

As shown in **Supplementary Table 14** and **Figure 5**, the ranking of feature importance is highly consistent across tasks, with Spearman’s rank correlation coefficients ranging from 0.955 to 0.959 for all task pairs. Furthermore, 12 acoustic features consistently appeared in the Top 20 lists across all four tasks (**Supplementary Table 14**), with spectral features (8), ZCR features (3), and high-frequency Mel spectral features (1) constituting a stable core set. The Jaccard similarity of the Top 20 features across spontaneous tasks (T2–T3: 0.818; T2–T4: 0.739; T3–T4: 0.600) was higher than that between reading tasks and spontaneous tasks (0.48–0.54), which is consistent with the superior screening performance observed for spontaneous tasks in Section 3.2.1.

**Figure 5:**
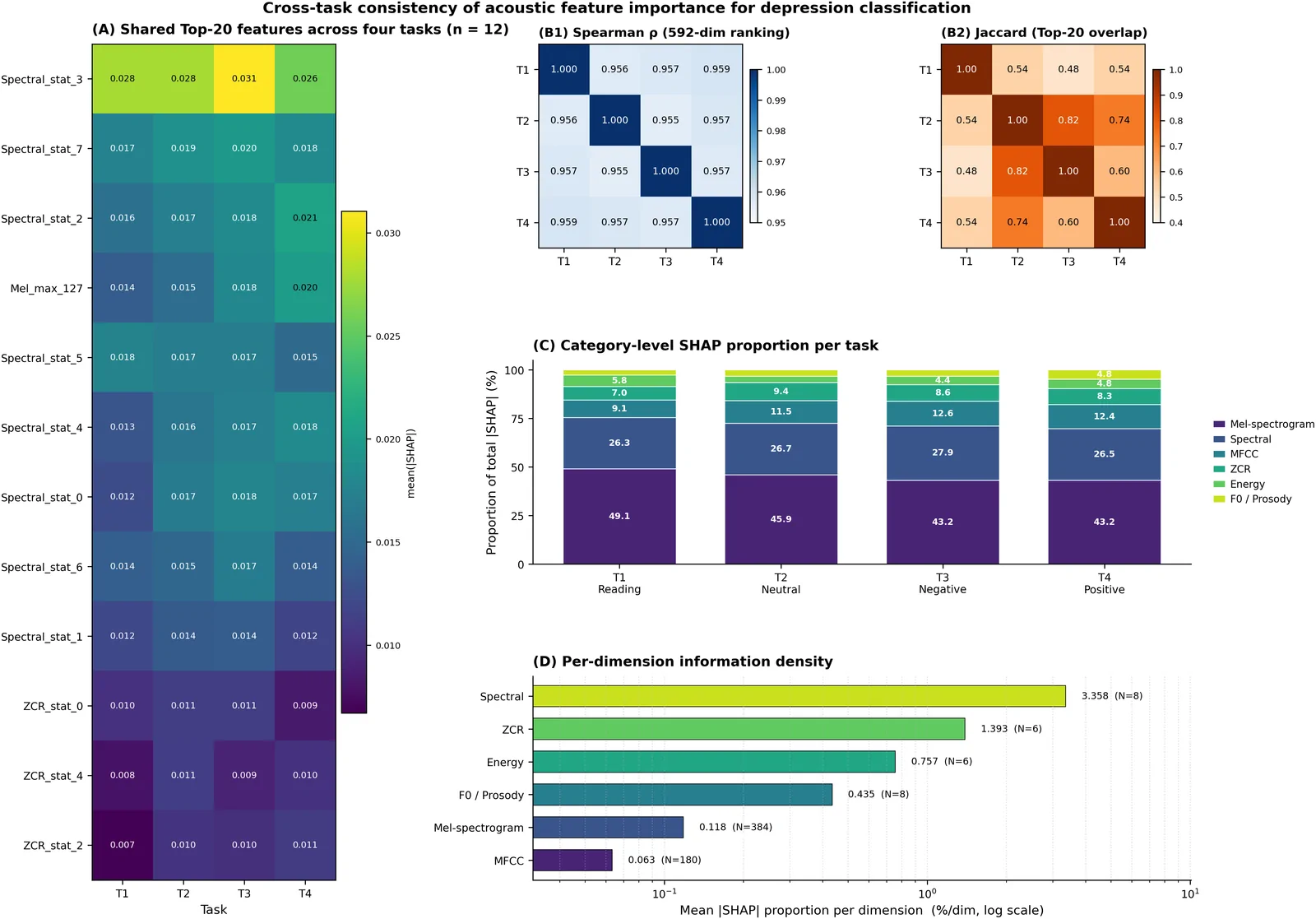
Cross-task consistency of acoustic feature importance for depressive symptoms classification. **(A)** Shared Top-20 features across four tasks (n = 12). **(B1)** Spearman correlation coefficients (592-dim ranking). **(B2)** Jaccard similarity (Top-20 overlap). **(C)** Category-level SHAP proportion per task. **(D)** Per-dimension information density.

In terms of feature categories, spectral features and Mel-spectral features collectively accounted for approximately 70–75% of the total SHAP contribution (**Supplementary Table 14**). F0 and prosodic statistics contributed 2.7–4.8%, while ZCR and energy features made moderate contributions that remained consistently stable across tasks. These findings are consistent with the existing literature, which links depression to changes in vocal resonance, narrowed pitch range, and psychomotor retardation. The high consistency in feature importance rankings across tasks confirms that the model detects stable, clinically meaningful acoustic correlates of depressive symptoms rather than task-specific artifacts.

### 3.3 Duration analysis and KL divergence: The “1-second golden window” and temporal redundancy

#### 3.3.1 Performance stability across time windows

When using the model to investigate the lower bound of speech duration, we made a counterintuitive discovery: in spontaneous speech tasks (T2/T3/T4), the AUC for windows ranging from 1 to 35 seconds remained stable at 0.810–0.833. Specifically, in Positive recall task(T4), the AUC for the 1-second window was 0.833, representing a decrease of only 3.14% compared to the full-duration AUC (0.860). We define this as the “1-second golden window” for depressive symptom screening.

#### 3.3.2 Cross-task stability and differences across task types

To verify the stability of the “1-second golden window,” we conducted cross-task validation on other tasks. The results indicate that this phenomenon is highly consistent across all spontaneous speech tasks (**Supplementary Table 9**; **Figures 6**, **7**).

**Figure 6:**
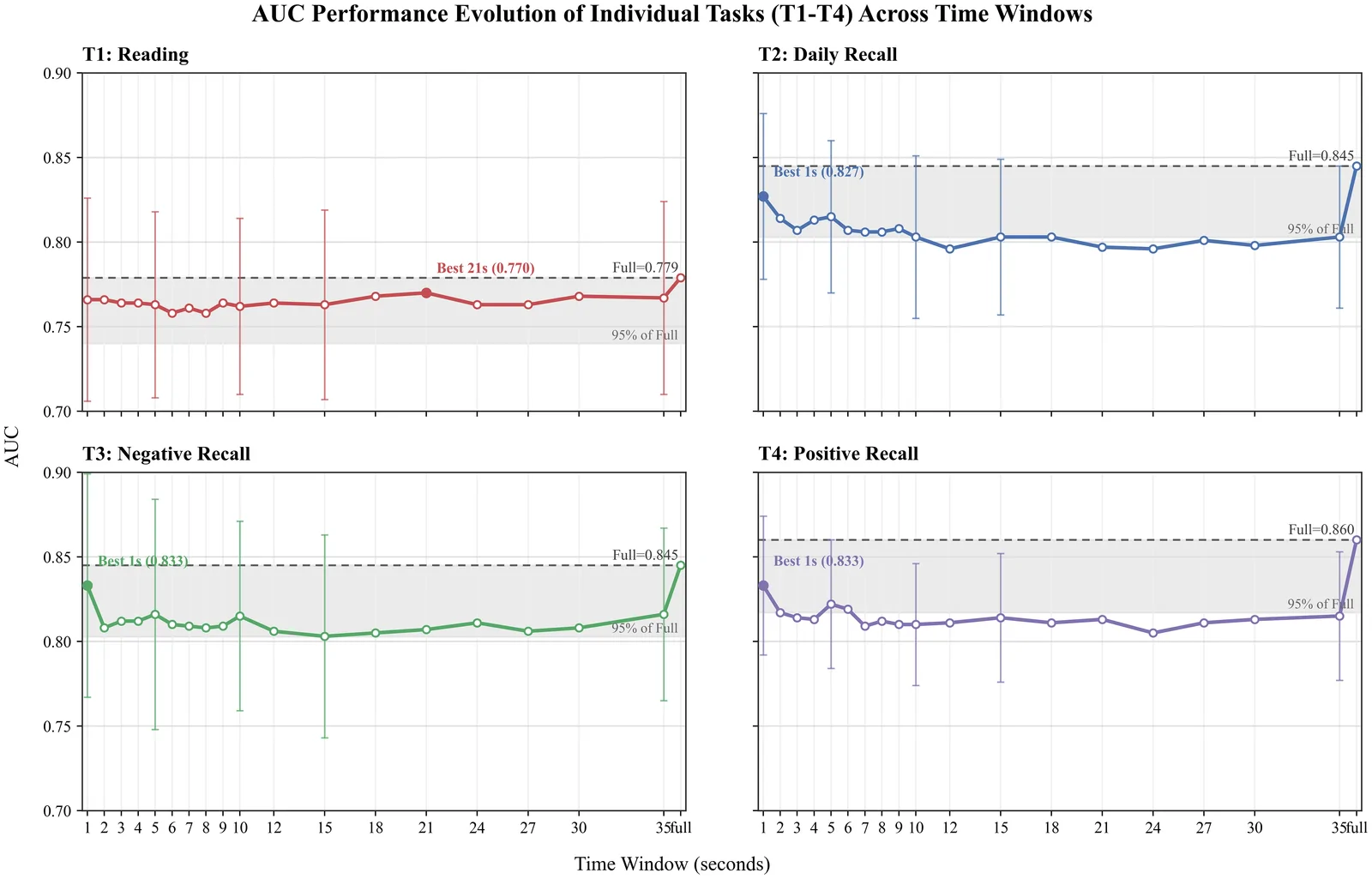
AUC performance of individual tasks (T1–T4) across different time windows.

**Figure 7:**
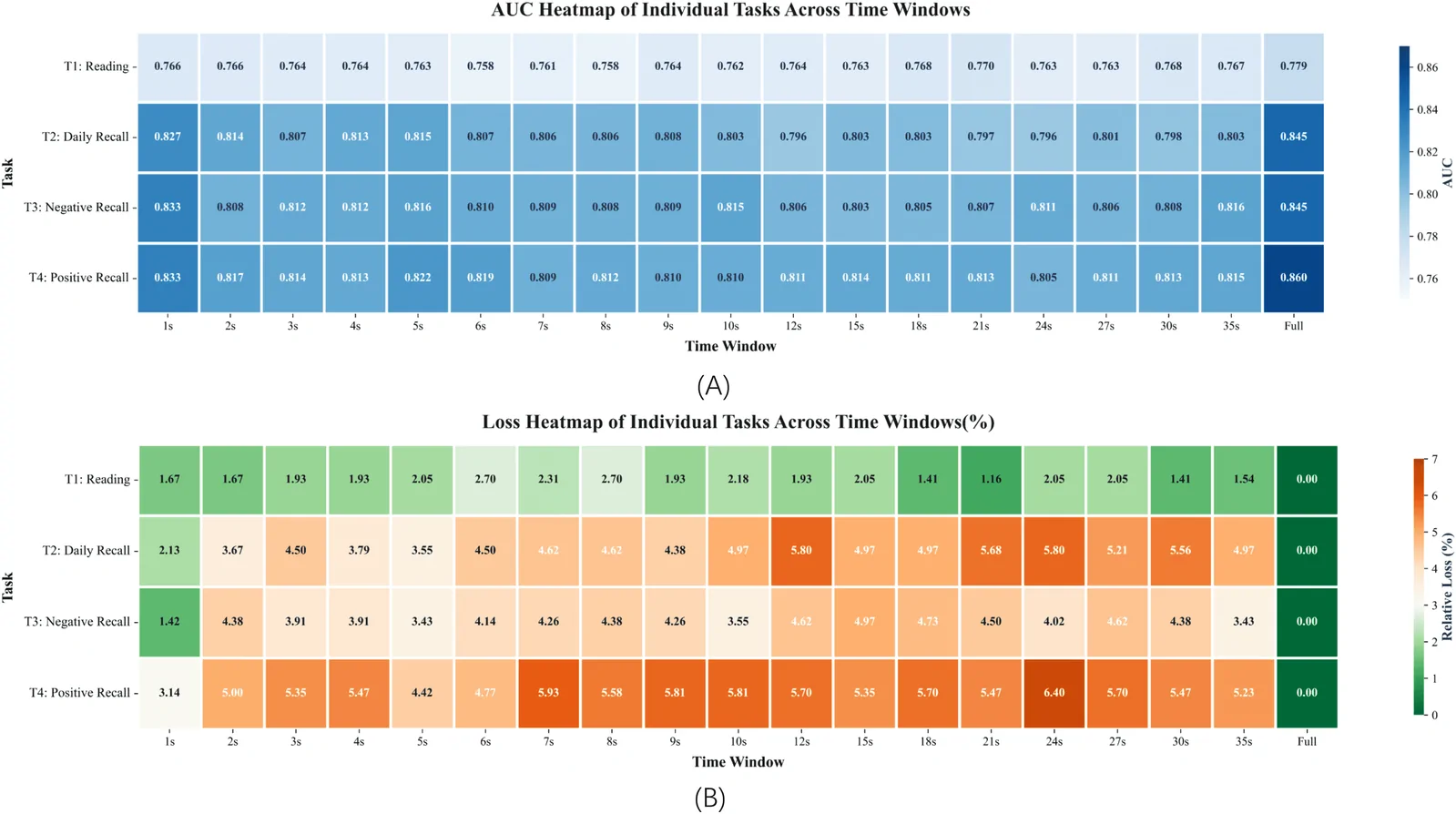
AUC and relative loss of individual tasks across different time windows. **(A)** Heatmap of AUC values for individual tasks across time windows. **(B)** Heatmap of relative loss (%) for individual tasks across time windows.

In the daily recall task (T2), the AUC for the 1-second window was 0.827, with a relative loss of only 2.13%. In the negative recall task (T3), the AUC for the 1-second window reached 0.833, with a relative loss of only 1.42%. This confirms that in spontaneous speech tasks, longer speech segments do not provide significant additional information, indicating that emotional speech exhibits high temporal redundancy.

However, task type influences the optimal duration. Since structured reading tasks may reduce speech differences across different populations, the optimal window for the T1 reading task was extended to 21 seconds (AUC = 0.770, loss 1.16%).

#### 3.3.3 KL divergence

The KL divergence quantifies the difference in acoustic distributions between truncated windows and the full duration. For T2, T3, and T4, the KL divergence of the 1-second window is the smallest among all time windows (9.99, 13.84, and 9.56, respectively), indicating that the acoustic feature distribution of the 1-second window is closest to that of the full duration (**Supplementary Table 10**).

Additionally, the AUC values of these windows are also closest to the AUC of the full duration. This suggests that extremely short time windows (1 second) share highly similar speech distribution characteristics with the full duration, effectively representing the core acoustic features of the entire speech while avoiding interference from macrosyntactic variations (such as pauses, syntactic structure, and emotional intonation) in subsequent speech segments. Consequently, the model can focus on micro-acoustic anomalies directly associated with depressive symptoms.

To further validate this conclusion, we additionally calculated the Fréchet distance (FAD) in the same feature space. As shown in **Supplementary Table S10** in the appendix, the FAD monotonically decreases with increasing window length across all tasks; the FAD value is highest for a 1-second window, while the KL divergence reaches its minimum at this point. This apparent discrepancy stems from the different mathematical properties of the two metrics: KL divergence captures differences in probability density distributions, whereas FAD measures geometric differences between multivariate Gaussian distributions in high-dimensional space and is more sensitive to covariance estimates. The limited sample size in short segments leads to an underestimation of covariance and an amplification of FAD. Despite this difference, both metrics point to the same conclusion: the 1-second window marks the inflection point at which classification performance saturates (AUC for T4 = 0.833, with a relative AUC loss of only 3.14% compared to the full duration). Therefore, although short clips appear geometrically deviated from the full distribution, they retain sufficient task-relevant information for effective screening.

It is also worth clarifying that KL divergence is used here as a relative metric for internal comparison rather than as an absolute measure. Its absolute magnitude depends on the feature space and has no universal threshold. Its utility lies in ranking window lengths by their proximity to the full recording, providing a complementary perspective to AUC. In this context, the key finding is that the 1-second window consistently achieves the smallest KL divergence among all short windows across spontaneous tasks, consistent with its near-optimal AUC.

### 3.4 Validation on the EATD dataset

To assess the robustness of the above results across datasets, we conducted a validation on the EATD dataset. Since the EATD dataset contains only neutral, negative, and positive recall tasks, the analysis was limited to the available tasks T1–T3 (T1: neutral; T2: negative; T3: positive).

First, when applying the same 592-dimensional features as in the main dataset, the classification performance was limited. For full-length recordings, the AUC values for tasks T1–T3 were 0.587, 0.535, and 0.528, respectively. Under this feature set, the optimal time window exhibited task-dependent differences: T1 reached its peak AUC at full length, while T2 and T3 achieved their maximum AUC at a 4-second truncation. Although the advantages of short windows were not entirely consistent, the effectiveness of temporal truncation remained significant in tasks T2 and T3.

To mitigate the feature space mismatch issue, a 2,256-dimensional feature set optimized for the acoustic characteristics of EATD was subsequently deployed (**Supplementary Table 11**). Under these conditions, the optimal time scale also exhibited significant task dependency: the T3 rapidly reached its peak at a 1-second truncation (AUC = 0.656), whereas T2 and T1 required 8 seconds (AUC = 0.663) and 15 seconds (AUC = 0.641), respectively, to reach their maxima. This confirms that in spontaneous speech tasks, very short time windows (e.g., 1 second) can effectively extract acoustic features capable of distinguishing depressive symptom, fully consistent with the findings from the main dataset.

Regarding task fusion at the 1-second cutoff, the standalone T3 task (AUC = 0.656) outperformed all multi-task combination configurations. This evaluates the absence of synergy between tasks, fully consistent with the findings from the main dataset (**Supplementary Table 12**).

Ultimately, the validation results of EATD confirm the following three core trends, which are highly consistent with the findings from the main dataset: first, task specificity, meaning that the optimal truncation window varies across different tasks; second, the discriminative advantage of extremely short windows in spontaneous speech, whereby in spontaneous speech tasks (especially positive recall), a 1-second truncation suffices to achieve optimal performance; third, the redundancy of multi-task fusion, meaning there is no synergy among the tasks. Although absolute performance across datasets is sensitive to feature engineering, the enhanced representation method robustly reproduces these three core trends.

The performance drop on EATD can be attributed to differences in recording conditions, task design, and labeling procedures between datasets, which introduce distribution shifts when features are applied without adaptation.

### 3.5 Statistical analysis

To assess the statistical reliability of the core comparison results and address the issue of multiple comparisons, we conducted formal hypothesis tests for all main experiments. All experiments employed a speaker-independent classification approach and were repeated five times using different random seeds. For each comparison, we extracted performance metrics from the five repetitions and performed paired tests (using a paired t-test when the normality assumption was met, and the Wilcoxon signed-rank test otherwise). To control the false discovery rate resulting from multiple comparisons, we employed the Benjamini-Hochberg method; corrected p-values (q-values) < 0.05 were considered statistically significant. Analysis of multi-task synergy effects utilized bootstrap confidence intervals and permutation tests. A detailed summary of all statistical comparisons is provided in **Supplementary Table 13**.

Comparison by task: T4 (Positive recall task, AUC = 0.860 ± 0.032) was significantly superior to T1 (reading task, AUC = 0.779 ± 0.062; Cohen’s d = 1.64, 95% CI [0.052, 0.110], q < 0.001). However, the performance differences between T4 and T2 (AUC = 0.845 ± 0.053) and between T4 and T3 (AUC = 0.845 ± 0.069) were not statistically significant (q = 0.303 for both). This indicates that the performance of all spontaneous emotional tasks (T2, T3, T4) is at the same level, while the discriminatory power of the structured reading task (T1) is significantly weaker.

Time-Window Analysis: Although statistically significant differences were observed between the 1-second window and the full-length recording across all tasks (all q < 0.05), the absolute performance losses were small: 1.67% for T1, 2.13% for T2, 1.42% for T3, and 3.14% for T4. These results indicate that the statistical significance is primarily driven by the high precision of repeated measurements, while the actual performance differences are minimal. From an application perspective, a 1-second speech sample is sufficient to support low-burden screening applications.

multi-task synergy: None of the multi-task combinations produced a synergistic gain significantly superior to that of the best single-task T4. The 95% confidence intervals for the synergy indices of all combinations included zero, and all q-values were greater than 0.05 after FDR correction, confirming the absence of multi-task synergy.

Feature-Model Matching: A comparison between frame-level features + Transformer (optimal window: 2 seconds) and statistical features + Transformer (optimal window: 10 seconds) showed no significant difference in AUC (mean difference = +0.21, Cohen’s d = 0.23, 95% CI [-0.121, 0.174], q = 0.935). This suggests that the “window compensation” mechanism of sequence models for statistical features is more of a trend than a statistically robust effect; future research should further validate this finding with larger samples.

## 4 Discussion

This study systematically investigates three core issues in acoustic-based depressive symptom screening: task selection, minimum speech duration, and feature-model matching.

### 4.1 Short-window characteristics of depressive speech features

We found that a 1-s speech segment can achieve screening performance for depressive symptoms that is comparable to that obtained from the full recording (for T4, AUC = 0.833, with only a 3.14% performance loss), compressing the previously reported “10-s effective window” (44) down to 1 s. KL divergence analysis showed that the feature distribution of the 1-second window was highly consistent with the global distribution, implying that screening can be completed using just a 1-second speech sample, provided that the features and model are properly matched. Furthermore, a 1-second speech segment contains less semantic noise, thereby highlighting acoustic-level variations more clearly (45).

In addition, screening performance peaked at 1 s and then declined as the window length increased, suggesting a possible “transient” nature of depressive speech markers. Participants with depressive symptoms appear to exhibit the most pronounced psychomotor retardation in the very first second of speech, whereas subsequent segments increasingly incorporate macro-linguistic noise (e.g., cognitive pauses and higher-order linguistic structure), which can obscure the core acoustic signatures. This observation is consistent with earlier findings that “short segments of speech can still provide accurate classification decisions” (46). Together, these results not only support the principle that “less is more” in screening, but also provide an acoustic foundation for applying the psychological “thin-slicing” framework (47) to speech-based depressive symptom screening.

### 4.2 Single-task superiority and zero-synergy in multi-task fusion

The study found that the single positive recall task (T4) yielded the best performance (AUC = 0.860), outperforming all multi-task combinations. Spontaneous speech tasks (T2/T3/T4) all performed better than the reading task (T1), confirming that spontaneous speech has higher ecological validity (48). Furthermore, the combination of all tasks (AUC = 0.833) not only failed to produce synergistic gains but actually triggered a phenomenon of “negative transfer” (49, 50).

Synergy analysis indicates that blindly combining tasks leads to information redundancy, highlighting the principle that “carefully selecting a single high-value task” is superior to “blindly combining multiple tasks” in speech screening for depressive symptoms. This provides clear methodological guidance for developing low-burden, rapid screening tools. A screening protocol requiring only a 1-second voice sample can be directly integrated into mobile or telemedicine systems, offering a viable solution to meet the need for objective screening in settings with limited medical resources.

### 4.3 Coupling between features, models, and window lengths

Reference (51) demonstrated that Transformers can achieve state-of-the-art performance in affective computing when processing highly fine-grained acoustic signals, but did not explicitly delineate the temporal boundaries of this advantage. Our findings further clarify the underlying “feature–model–window” coupling rules: frame-level features need to be paired with sequence models and short windows (e.g., Transformer reaching 73.11% accuracy at 2 s), whereas statistical features generally favored tree-based models; for Random Forest, the optimal window shortened to 1 s, whereas sequence models tended to require longer windows.

Moreover, the “forced window extension” phenomenon observed in our study—where the optimal window for the Transformer abruptly increased from 2 s to 10 s when fed with purely statistical features—provides a counterexample to a simplistic interpretation of (50). Mechanistically, it suggests that the advantage of sequence models fundamentally depends on sub-second, micro-temporal modeling. This not only explains why cutting-edge sequence models are best paired with high-resolution acoustic features, but also offers clear design principles for model architectures and temporal slicing in future speech-based depressive symptom screening systems.

In addition, under the 1-s segmentation setting, expanding the feature set to 592 dimensions by introducing global statistics (mean, standard deviation, and maximum of the 188 frame-level features, plus 28 statistical features) increased the AUC of Random Forest to 0.833. As noted in (52), such “global features” effectively condense macro-level statistical information. In our context, these high-dimensional statistics compensate for the limited temporal modeling capacity of tree-based models and substantially improve classification performance.

### 4.4 Implications for digital mental health

The above findings provide a basis for digital tools for the remote screening of depressive symptoms. According to the experimental results, the use of short voice recordings reduces the burden on participants. It also allows the model to be deployed on web or mobile platforms for large-scale population screening in settings such as community clinics and schools. This offers a feasible implementation plan for large-scale mental health screening and enriches the array of digital mental health screening tools. However, it is important to emphasize that such systems should be used as supplementary tools and cannot replace a physician’s diagnosis.

### 4.5 Limitations and future directions

The sample in this study consisted of individuals with mild to moderate depressive symptoms (PHQ-9 scores of 5-14). A major limitation is that clinical diagnoses were not made using structured interviews based on the DSM-5. Therefore, the findings of this study should be interpreted as acoustic features associated with depressive symptoms rather than directly extrapolated to clinically diagnosed patients with depression; further validation in patients with severe depression is required. Another consideration is that the data were collected at a single center in Hainan, China. Although the results have been evaluated using the EATD dataset, cross-linguistic validation remains necessary.

It is also important to reiterate that our contribution is not a generalizable, off-the-shelf screening model, but rather a set of evidence-based design principles. The EATD validation supports this framing: while absolute performance was sensitive to feature representation, the three core trends, task specificity, the advantage of short windows in spontaneous speech, and the absence of multi-task synergy, remained consistent across datasets, suggesting that the principles generalize even when absolute performance does not.

Future research will focus on validation in patients with moderate-to-severe depression, cross-cultural validation, and integration with other modalities.

## 5 Conclusion

Rather than pursuing a clinically deployable detector, this study provides evidence-based design principles for constructing low-burden screening protocols by systematically deconstructing three fundamental issues: task selection, minimum speech duration, and feature-model matching. The core findings that support these principles are summarized as follows:

1. At the task level: The positive recall task (T4) achieved the best overall performance (AUC = 0.860) among all single tasks and multi-task combinations, outperforming all other single tasks and multi-task combinations; the multi-task combinations did not exhibit synergy.
2. At the temporal window level: Taking T4 as an example, a 1-second segment achieves AUC comparable to that of the full duration (relative loss = 3.14%). The KL divergence (9.56) confirms that the feature distribution of the short window differs only slightly from that of the full speech, while eliminating macrosyntactic noise.
3. At the feature–model level: Frame-level features need to be paired with sequence models and short windows (0.1–2 s), with the Transformer reaching an accuracy of 0.731 at 2 s. Fused features further improved tree-based models, with Random Forest reaching 0.703 accuracy at 4 s. The observed “forced window extension” phenomenon (Transformer’s optimal window shifting from 2 s to 10 s with statistical features) further underscores the fundamental reliance of sequence models on micro-temporal dynamics.

Based on these findings, we propose two standardized and deployable screening protocols:

1. A 2-second high-accuracy protocol (Positive recall task + 2-s truncation + frame-level features + Transformer), suitable for scenarios that prioritize maximal performance; and
2. A 1-second resource-efficient protocol (Positive recall task + 1-s truncation + statistical features + Random Forest), suitable for resource-constrained settings or contexts in which model interpretability is paramount.

In addition, the supplementary analysis on EATD showed that absolute cross-dataset performance was sensitive to feature representation, and generalization under the strictly aligned reference setting remained limited. Under the enhanced statistical aggregation representation, however, the three core findings—task-specific differences, the advantage of short time windows, and the absence of multi-task synergy—were further supported. These results suggest that the core findings of the present study are reasonably robust, although their external generalizability still requires further validation.

In summary, this study demonstrates that the use of short-window, task-specific speech samples can effectively support low-burden screening. The “task, time-window, feature, and model” approach proposed in this paper can serve as a reference for the design of future digital mental health screening tools; however, it is important to clarify that its role remains that of an auxiliary screening tool rather than a clinical diagnostic tool. Further validation of its effectiveness under specific clinical conditions is required.

Importantly, the contribution of this study is not a generalizable, off-the-shelf screening model, but rather a set of evidence-based design principles for constructing low-burden screening protocols. These principles are intended to guide the development of screening tools in new datasets and settings, rather than to serve as a one-size-fits-all solution.

## Data Availability

The datasets presented in this article are not readily available because they contain potentially sensitive information from human participants, including speech recordings and questionnaire data, and are therefore subject to privacy, ethical, and institutional data-sharing restrictions. Requests to access the datasets should be directed to Lijuan Liang (hy0203043@muhn.edu.cn). The publicly available EATD dataset can be obtained from its original source as described in the original publication.
