# Supplementary material for: Low-burden speech-based screening of depressive symptoms using ultra-short speech samples
Source: Front Psychiatry. 2026 Sep 23;17:1888425. doi: 10.3389/fpsyt.2026.1888425 (PMC13643431; doi:10.3389/fpsyt.2026.1888425)
Supplement: Supplementary file 1 [file Table1.docx]

**Table S1.** Audio feature dimensionality summary.

| **Feature Type** | **Subtype** | **Dimension** | **Description** |
| --- | --- | --- | --- |
| Frame-Level Features (188 dimensions) | Mel-Spectrogram Features | 128 | Log-Mel spectrogram extracted using 128 Mel filter banks (n_mels = 128) |
|  | Enhanced MFCC Features | 60 | Base MFCC: 20 +delta: 20 +delta-delta: 20 |
| Statistical Features (28 dimensions) | F0 (pitch) Statistics | 8 | Mean, standard deviation, median, 25th/75th percentiles, voiced segment ratio, skewness, kurtosis |
|  | Zero-Crossing Rate (ZCR) Statistics | 6 | Mean, standard deviation, median, 25th/75th percentiles, skewness |
|  | Short-Time Energy Statistics | 6 | Mean, standard deviation, median, 25th/75th percentiles, skewness |
|  | Spectral Statistics | 8 | Spectral centroid, bandwidth, roll-off, and flatness (mean + std for each) |
| Total | - | **216** **dimensions** | 188 frame-level + 28 statistical features |

**Table S2.** Model architecture and training hyperparameters.

| **Component** | **Parameter** | **Value & Description** |
| --- | --- | --- |
| **Input Settings** | Input Feature Type | 216 dimensions (188 frame-level + 28 statistical features) |
|  | Output Tasks | Classification (2 classes: HC vs. Depressive symptoms) |
| **Random Forest** | Architecture | Ensemble of 300 decision trees |
|  | Tree Parameters | Max depth: None, Min samples split: 10, Min samples leaf: 5 |
|  | Feature Engineering | PCA (reduced to 20 principal components) |
| **XGBoost** | Architecture | Gradient boosting framework with 100 boosting rounds |
|  | Tree Parameters | Max depth: 6, Learning rate: 0.1, Subsample: 0.8, Colsample: 0.8 |
|  | Feature Engineering | PCA(0.95) |
| **LSTM** | Architecture | 2-layer unidirectional LSTM |
|  | Hidden Size | 128 |
|  | Regularization | LSTM dropout: 0.5, Feature dropout: 0.3 |
|  | Training Settings | Batch size: 128, Optimizer: Adam (lr=3e-4, weight_decay=5e-4) |
| **Transformer** | Architecture | 2-layer standard Transformer Encoder |
|  | Attention & FFN | 4 attention heads, d_model = 128, FFN dimension = 256 |
|  | Regularization | Dropout: 0.1, Gradient clipping (max norm = 0.5) |
|  | Training Settings | Batch size: 256, Optimizer: Adam (lr=5e-5, weight_decay=1e-2) |
| **xLSTM** | Architecture | 2 layers (alternating sLSTM and mLSTM blocks) |
|  | Hidden Size & Heads | Hidden dimension: 60, Attention heads: 5 |
|  | Regularization | Dropout: 0.1, Gradient clipping (max norm = 0.5) |
|  | Training Settings | Batch size: 128, Optimizer: AdamW (lr=5e-4, weight_decay=1e-2) |
|  | LR Scheduler | ReduceLROnPlateau (patience=8, factor=0.5) |
| **Common Training** | Max Epochs | 100 epochs for all deep learning models |
| **Settings (DL)** | Early Stopping | Patience = 15 epochs (monitored on validation loss) |

**Note:** All hyperparameters were fixed (see Section 2.5) and are reported as implemented in each model. The cross-validation and data splitting procedures are described in Section 2.5.

**Table S3.** Multi-task types and comprehensive combinatorial ablation experimental schemes.

| **Experiment Type** | **Experiment ID** | **Task Combination** | **Task Description** |
| --- | --- | --- | --- |
| Full-task model | A | T1+T2+T3+T4 | Combines neutral, daily, positive and negative speech tasks |
| Single Task | T1 | T1 | Neutral reading (contains only standardized, neutral emotional speech tasks). |
|  | T2 | T2 | Daily conversation (naturalistic daily interaction speech task) |
|  | T3 | T3 | Negative recall (speech task under negative emotional arousal) |
|  | T4 | T4 | Positive recall (speech task under positive emotional arousal) |
| Leave-One-Out | C1 | T2 + T3 + T4 | Excluding T1: assesses contribution of standardized neutral baseline to the full model |
|  | C2 | T1 + T3 + T4 | Excluding T2: assesses contribution of natural daily state to the full model |
|  | C3 | T1 + T2 + T4 | Excluding T3: assesses contribution of negative emotional features to the full model |
|  | C4 | T1 + T2 + T3 | Excluding T4: assesses contribution of positive emotional features to the full model |
| Two-Task Combinations | D1 | T1 + T3 | Neutral-negative contrast: evaluates joint effect of standardized baseline and negative emotional arousal |
|  | D2 | T2 + T3 | Daily-negative combination: evaluates feature complementarity between natural interaction and negative emotion |
|  | D3 | T3 + T4 | Emotional polarity contrast: pure emotion-induction task combination, validates joint classification efficacy of positive and negative emotional valence |
|  | D4 | T1 + T2 | Non-emotional baseline combination: evaluates basic speech features without significant emotional induction |
|  | D5 | T1 + T4 | Neutral-positive contrast: evaluates joint effect of standardized baseline and positive emotional arousal |
|  | D6 | T2 + T4 | Daily-positive combination: evaluates feature complementarity between natural interaction and positive emotion |

**Table S4.** Optimal performance under 188-dimensional frame-level features.

| **Model** | **Optimal Window** | **Accuracy** | **Specificity** | **Precision** | **F1-Score** | **AUC** | **Recall** |
| --- | --- | --- | --- | --- | --- | --- | --- |
| RandomForest | 4 s | 0.584 ± 0.016 | 0.706 ± 0.030 | 0.584 ± 0.016 | 0.577 ± 0.019 | 0.628 ± 0.019 | 0.570 ± 0.022 |
| XGBoost | 4 s | 0.575 ± 0.031 | 0.739 ± 0.019 | 0.573 ± 0.019 | 0.561 ± 0.037 | 0.605 ± 0.016 | 0.549 ± 0.044 |
| Transformer | 2 s | 0.731 ± 0.030 | 0.675 ± 0.077 | 0.664 ± 0.041 | 0.722 ± 0.042 | 0.798 ± 0.036 | 0.791 ± 0.063 |
| LSTM | 1 s | 0.722 ± 0.030 | 0.685 ± 0.041 | 0.660 ± 0.036 | 0.708 ± 0.041 | 0.795 ± 0.029 | 0.764 ± 0.059 |
| xLSTM | 0.1 s | 0.726 ± 0.016 | 0.735 ± 0.023 | 0.752 ± 0.021 | 0.724 ± 0.019 | 0.777 ± 0.026 | 0.698 ± 0.027 |

**Table S5. Optimal** performance under 28-dimensional statistical features.

| **Model** | **Optimal window** | **Accuracy** | **Specificity** | **Precision** | **F1-score** | **AUC** | **Recall** |
| --- | --- | --- | --- | --- | --- | --- | --- |
| RandomForest | 1 s | 0.698 ± 0.032 | 0.669 ± 0.045 | 0.707 ± 0.039 | 0.699 ± 0.033 | 0.770 ± 0.033 | 0.691 ± 0.044 |
| XGBoost | 5 s | 0.664 ± 0.054 | 0.677 ± 0.055 | 0.673 ± 0.055 | 0.665 ± 0.055 | 0.730 ± 0.056 | 0.658 ± 0.072 |
| LSTM | 2 s | 0.705 ± 0.026 | 0.676 ± 0.067 | 0.649 ± 0.034 | 0.689 ± 0.027 | 0.777 ± 0.030 | 0.734 ± 0.039 |
| Transformer | 10 s | 0.700 ± 0.050 | 0.708 ± 0.061 | 0.666 ± 0.051 | 0.676 ± 0.049 | 0.771 ± 0.055 | 0.687 ± 0.064 |
| xLSTM | 15 s | 0.697 ± 0.050 | 0.696 ± 0.049 | 0.701 ± 0.050 | 0.697 ± 0.050 | 0.754 ± 0.062 | 0.693 ± 0.064 |

**Table S6.** Optimal performance under 216-dimensional fused features.

| **Model** | **Optimal window** | **Accuracy** | **Specificity** | **Precision** | **F1-score** | **AUC** | **Recall** |
| --- | --- | --- | --- | --- | --- | --- | --- |
| RandomForest | 4 s | 0.703 ± 0.040 | 0.672 ± 0.064 | 0.712 ± 0.044 | 0.704 ± 0.041 | 0.767 ± 0.050 | 0.696 ± 0.052 |
| XGBoost | 4 s | 0.682 ± 0.040 | 0.694 ± 0.063 | 0.685 ± 0.041 | 0.683 ± 0.040 | 0.751 ± 0.048 | 0.681 ± 0.051 |
| LSTM | 1 s | 0.700 ± 0.026 | 0.690 ± 0.069 | 0.647 ± 0.026 | 0.674 ± 0.035 | 0.774 ± 0.032 | 0.703 ± 0.045 |
| Transformer | 1 s | 0.709 ± 0.037 | 0.683 ± 0.053 | 0.651 ± 0.043 | 0.692 ± 0.038 | 0.788 ± 0.043 | 0.739 ± 0.054 |
| xLSTM | 0.1 s | 0.722 ± 0.015 | 0.729 ± 0.019 | 0.742 ± 0.016 | 0.721 ± 0.018 | 0.779 ± 0.027 | 0.701 ± 0.024 |

**Table S7.** Single-task performance comparison.

| **Task** | **AUC** | **Accuracy** | **Specificity** | **Precision** | **F1-Score** |
| --- | --- | --- | --- | --- | --- |
| Task1(T1) | 0.779 ± 0.062 | 0.731 ± 0.055 | 0.606 ± 0.102 | 0.683 ± 0.115 | 0.756 ± 0.071 |
| Task2(T2) | 0.845 ± 0.053 | 0.739 ± 0.063 | 0.836 ± 0.077 | 0.699 ± 0.138 | 0.633 ± 0.074 |
| Task3(T3) | 0.845 ± 0.069 | 0.760 ± 0.084 | 0.807 ± 0.085 | 0.728 ± 0.092 | 0.712 ± 0.064 |
| Task4(T4) | **0.860 ± 0.032** | 0.749 ± 0.039 | 0.853 ± 0.061 | 0.713 ± 0.117 | 0.639 ± 0.037 |

**Table S8.** Multi-task combinations and their performance metrics.

| **Exp. ID** | **Task Combination** | **AUC** | **Accuracy** | **Specificity** | **Precision** | **F1 Score** | **Change vs T4 (AUC)** | **Highest Single‑Task AUC** | **Synergy Index** | **Synergy Effect** |
| --- | --- | --- | --- | --- | --- | --- | --- | --- | --- | --- |
| T4 | T4 | 0.860 ± 0.032 | 0.749 ± 0.039 | 0.853 ± 0.061 | 0.713 ± 0.117 | 0.639 ± 0.037 | Baseline | 0.860 | — | — |
| D6 | T2 + T4 | 0.859 ± 0.057 | 0.748 ± 0.048 | 0.840 ± 0.093 | 0.704 ± 0.172 | 0.643 ± 0.075 | -0.001 | 0.860 | -0.001 | No synergy |
| C1 | T2 + T3 + T4 | 0.859 ± 0.051 | 0.765 ± 0.063 | 0.813 ± 0.080 | 0.716 ± 0.111 | 0.705 ± 0.053 | -0.001 | 0.860 | -0.001 | No synergy |
| D2 | T2 + T3 | 0.855 ± 0.035 | 0.758 ± 0.055 | 0.811 ± 0.051 | 0.704 ± 0.121 | 0.691 ± 0.059 | -0.005 | 0.845 | -0.010 | No synergy |
| D3 | T3 + T4 | 0.853 ± 0.048 | 0.761 ± 0.054 | 0.826 ± 0.063 | 0.715 ± 0.096 | 0.684 ± 0.052 | -0.007 | 0.860 | -0.007 | No synergy |
| T3 | T3 | 0.845 ± 0.069 | 0.760 ± 0.084 | 0.807 ± 0.085 | 0.728 ± 0.092 | 0.712 ± 0.064 | -0.015 | 0.845 | — | — |
| T2 | T2 | 0.845 ± 0.053 | 0.739 ± 0.063 | 0.836 ± 0.077 | 0.699 ± 0.138 | 0.633 ± 0.074 | -0.015 | 0.845 | — | — |
| A | T1 + T2 + T3 + T4 | 0.833 ± 0.046 | 0.753 ± 0.043 | 0.732 ± 0.079 | 0.698 ± 0.106 | 0.736 ± 0.044 | -0.027 | 0.860 | -0.027 | No synergy |
| C2 | T1 + T3 + T4 | 0.818 ± 0.048 | 0.752 ± 0.042 | 0.707 ± 0.074 | 0.698 ± 0.096 | 0.746 ± 0.054 | -0.042 | 0.860 | -0.042 | No synergy |
| C3 | T1 + T2 + T4 | 0.815 ± 0.070 | 0.745 ± 0.059 | 0.709 ± 0.100 | 0.692 ± 0.118 | 0.736 ± 0.059 | -0.045 | 0.860 | -0.045 | No synergy |
| C4 | T1 + T2 + T3 | 0.814 ± 0.071 | 0.757 ± 0.060 | 0.701 ± 0.109 | 0.706 ± 0.082 | 0.756 ± 0.049 | -0.046 | 0.845 | -0.031 | No synergy |
| D5 | T1 + T4 | 0.808 ± 0.052 | 0.739 ± 0.043 | 0.672 ± 0.096 | 0.689 ± 0.098 | 0.745 ± 0.044 | -0.052 | 0.860 | -0.052 | No synergy |
| D1 | T1 + T3 | 0.806 ± 0.060 | 0.748 ± 0.049 | 0.665 ± 0.096 | 0.698 ± 0.092 | 0.759 ± 0.050 | -0.054 | 0.845 | -0.039 | No synergy |
| D4 | T1 + T2 | 0.803 ± 0.070 | 0.741 ± 0.061 | 0.669 ± 0.108 | 0.688 ± 0.105 | 0.746 ± 0.065 | -0.057 | 0.845 | -0.042 | No synergy |
| T1 | T1 | 0.779 ± 0.062 | 0.731 ± 0.055 | 0.606 ± 0.102 | 0.683 ± 0.115 | 0.756 ± 0.071 | -0.081 | 0.779 | — | — |

Note**:** Synergy index = Combined AUC - Highest single-task AUC within the combination. An index close to zero suggests limited complementary information among the tasks, yielding no positive synergistic gain.

**Table S9.** AUC performance evolution of different speech tasks across time windows.

| **Time Window** | **Task 1: AUC (Cross)** | **Task 2: AUC (Cross)** | **Task 3: AUC (Cross)** | **Task 4: AUC (Cross)** |
| --- | --- | --- | --- | --- |
| 1 s | 0.766 ± 0.060 (↓1.67%) | **0.827 ± 0.049 (↓2.13%)** | **0.833 ± 0.066 (↓1.42%)** | **0.833 ± 0.041 (↓3.14%)** |
| 2 s | 0.766 ± 0.056 (↓1.67%) | 0.814 ± 0.048 (↓3.67%) | 0.808 ± 0.064 (↓4.38%) | 0.817 ± 0.031 (↓5.00%) |
| 3 s | 0.764 ± 0.057 (↓1.93%) | 0.807 ± 0.043 (↓4.50%) | 0.812 ± 0.068 (↓3.91%) | 0.814 ± 0.040 (↓5.35%) |
| 4 s | 0.764 ± 0.056 (↓1.93%) | 0.813 ± 0.047 (↓3.79%) | 0.812 ± 0.066 (↓3.91%) | 0.813 ± 0.038 (↓5.47%) |
| 5 s | 0.763 ± 0.055 (↓2.05%) | 0.815 ± 0.045 (↓3.55%) | 0.816 ± 0.068 (↓3.43%) | 0.822 ± 0.038 (↓4.42%) |
| 6 s | 0.758 ± 0.057 (↓2.70%) | 0.807 ± 0.044 (↓4.50%) | 0.810 ± 0.062 (↓4.14%) | 0.819 ± 0.040 (↓4.77%) |
| 7 s | 0.761 ± 0.057 (↓2.31%) | 0.806 ± 0.045 (↓4.62%) | 0.809 ± 0.061 (↓4.26%) | 0.809 ± 0.041 (↓5.93%) |
| 8 s | 0.758 ± 0.056 (↓2.70%) | 0.806 ± 0.045 (↓4.62%) | 0.808 ± 0.060 (↓4.38%) | 0.812 ± 0.037 (↓5.58%) |
| 9 s | 0.764 ± 0.052 (↓1.93%) | 0.808 ± 0.043 (↓4.38%) | 0.809 ± 0.062 (↓4.26%) | 0.810 ± 0.033 (↓5.81%) |
| 10 s | 0.762 ± 0.052 (↓2.18%) | 0.803 ± 0.048 (↓4.97%) | 0.815 ± 0.056 (↓3.55%) | 0.810 ± 0.036 (↓5.81%) |
| 12 s | 0.764 ± 0.054 (↓1.93%) | 0.796 ± 0.049 (↓5.80%) | 0.806 ± 0.054 (↓4.62%) | 0.811 ± 0.037 (↓5.70%) |
| 15 s | 0.763 ± 0.056 (↓2.05%) | 0.803 ± 0.046 (↓4.97%) | 0.803 ± 0.060 (↓4.97%) | 0.814 ± 0.038 (↓5.35%) |
| 18 s | 0.768 ± 0.055 (↓1.41%) | 0.803 ± 0.044 (↓4.97%) | 0.805 ± 0.058 (↓4.73%) | 0.811 ± 0.038 (↓5.70%) |
| 21 s | **0.770 ± 0.055 (↓1.16%)** | 0.797 ± 0.048 (↓5.68%) | 0.807 ± 0.054 (↓4.50%) | 0.813 ± 0.035 (↓5.47%) |
| 24 s | 0.763 ± 0.060 (↓2.05%) | 0.796 ± 0.046 (↓5.80%) | 0.811 ± 0.055 (↓4.02%) | 0.805 ± 0.038 (↓6.40%) |
| 27 s | 0.763 ± 0.060 (↓2.05%) | 0.801 ± 0.046 (↓5.21%) | 0.806 ± 0.055 (↓4.62%) | 0.811 ± 0.037 (↓5.70%) |
| 30 s | 0.768 ± 0.057 (↓1.41%) | 0.798 ± 0.043 (↓5.56%) | 0.808 ± 0.054 (↓4.38%) | 0.813 ± 0.040 (↓5.47%) |
| 35 s | 0.767 ± 0.057 (↓1.54%) | 0.803 ± 0.042 (↓4.97%) | 0.816 ± 0.051 (↓3.43%) | 0.815 ± 0.038 (↓5.23%) |
| Full | 0.779 ± 0.062 (-) | 0.845 ± 0.053 (-) | 0.845 ± 0.069 (-) | 0.860 ± 0.032 (-) |

**Table S10.** KL divergence of different speech tasks across time windows.

| **Time Window** | **Task 1** | | |  |  | **Task 2** | | | | **Task 3** | |  | **Task 4** | |
| --- | --- | --- | --- | --- | --- | --- | --- | --- | --- | --- | --- | --- | --- | --- |
|  | **KL** | **FAD(10¹⁰)** | | | **KL** | | **FAD(10¹⁰)** | | | **KL** | **FAD(10¹⁰)** | | **KL** | **FAD(10¹⁰)** |
| 1 s | 16.33 | 141.10 |  | | **9.99** | | 193.83 | |  | **13.84** | 208.85 |  | **9.56** | 200.57 |
| 2 s | 16.33 | 67.17 |  | | 13.72 | | 91.37 | |  | 16.19 | 96.15 |  | 13.67 | 94.88 |
| 3 s | 16.19 | 43.40 |  | | 14.63 | | 56.93 | |  | 16.04 | 58.64 |  | 14.38 | 59.42 |
| 4 s | 16.04 | 33.91 |  | | 14.30 | | 43.47 |  | | 15.95 | 43.88 |  | 13.97 | 45.07 |
| 5 s | 15.92 | 28.66 |  | | 15.48 | | 36.21 |  | | 15.85 | 35.96 |  | 17.03 | 37.25 |
| 6 s | 16.14 | 25.27 |  | | 15.52 | | 31.64 |  | | 16.04 | 30.94 |  | 15.08 | 32.09 |
| 7 s | 15.95 | 22.79 |  | | 15.52 | | 28.09 |  | | 15.89 | 27.35 |  | 15.04 | 28.35 |
| 8 s | 15.73 | 20.78 |  | | 15.43 | | 25.27 |  | | 15.66 | 24.67 |  | 14.97 | 25.39 |
| 9 s | 15.55 | 19.14 |  | | 15.39 | | 23.17 |  | | 15.52 | 22.48 |  | 14.89 | 23.05 |
| 10 s | 15.42 | 17.73 |  | | 15.29 | | 21.56 |  | | 15.38 | 20.68 |  | 14.80 | 21.11 |
| 12 s | 15.18 | 15.40 |  | | 15.15 | | 18.91 |  | | 15.16 | 17.86 |  | 14.65 | 18.18 |
| 15 s | 14.84 | 12.72 |  | | 14.92 | | 15.85 |  | | 14.88 | 14.90 |  | 14.39 | 15.06 |
| 18 s | 14.60 | 10.69 |  | | 14.74 | | 13.63 |  | | 14.70 | 12.73 |  | 14.21 | 12.76 |
| 21 s | 14.35 | 9.15 |  | | 14.57 | | 11.78 |  | | 14.53 | 11.05 |  | 14.06 | 10.99 |
| 24 s | 14.19 | 7.92 |  | | 14.48 | | 10.29 |  | | 14.37 | 9.68 |  | 13.92 | 9.70 |
| 27 s | 13.99 | 6.92 |  | | 14.40 | | 9.07 |  | | 14.18 | 8.50 |  | 13.80 | 8.58 |
| 30 s | 13.86 | 6.10 |  | | 14.28 | | 8.05 |  | | 14.07 | 7.51 |  | 13.71 | 7.67 |
| 35 s | 13.66 | 4.98 |  | | 14.11 | | 6.58 |  | | 13.92 | 6.17 |  | 13.59 | 6.33 |
| Full | 0.00 | 0.00 |  | | 0.00 | | 0.00 |  | | 0.00 | 0.00 |  | 0.00 | 0.00 |

**Table S11.** Full-length and best short-window results on the EATD dataset.

| **Feature Setting** | **Task** | | **Full AUC** | **Best Short Window** | **Best Short-window AUC** | **Relative Change** |
| --- | --- | --- | --- | --- | --- | --- |
| 2256-dim | T1 | 0.631 ± 0.151 | | 15 s | 0.641 ± 0.173 | +1.58% |
| 2256-dim | T2 | 0.521 ± 0.185 | | 8 s | 0.663 ± 0.144 | +27.26% |
| 2256-dim | T3 | 0.592 ± 0.126 | | 1 s | 0.656 ± 0.158 | +10.81% |
| 592-dim | T1 | 0.587 ± 0.082 | | 4 s | 0.546 ± 0.054 | -6.98% |
| 592-dim | T2 | 0.535 ± 0.085 | | 4 s | 0.577 ± 0.055 | +7.85% |
| 592-dim | T3 | 0.528 ± 0.087 | | 4 s | 0.528 ± 0.043 | +0.00% |

Note: For the EATD dataset, T1 = Neutral, T2 = Negative, and T3 = Positive.

**Table S12.** AUC comparison of EATD validation across representative time windows.

| **Feature Setting** | **Exp. ID** | **Task Combination** | **1 s AUC** | **5 s AUC** | **Full AUC** |
| --- | --- | --- | --- | --- | --- |
| 2256-dim | T1 | T1 | 0.386 ± 0.145 | 0.515 ± 0.144 | 0.631 ± 0.151 |
| 2256-dim | T2 | T2 | 0.610 ± 0.150 | 0.626 ± 0.166 | 0.521 ± 0.185 |
| 2256-dim | T3 | T3 | 0.656 ± 0.158 | 0.569 ± 0.159 | 0.592 ± 0.126 |
| 2256-dim | A | T1 + T2 + T3 | 0.590 ± 0.148 | 0.593 ± 0.158 | 0.593 ± 0.158 |
| 2256-dim | D1 | T1 + T2 | 0.500 ± 0.166 | 0.585 ± 0.183 | 0.585 ± 0.183 |
| 2256-dim | D2 | T1 + T3 | 0.539 ± 0.153 | 0.617 ± 0.158 | 0.617 ± 0.158 |
| 2256-dim | D3 | T2 + T3 | 0.649 ± 0.108 | 0.570 ± 0.145 | 0.570 ± 0.145 |
| 592-dim | T1 | T1 | 0.602 ± 0.154 | 0.584 ± 0.099 | 0.587 ± 0.082 |
| 592-dim | T2 | T2 | 0.408 ± 0.180 | 0.555 ± 0.073 | 0.535 ± 0.085 |
| 592-dim | T3 | T3 | 0.497 ± 0.189 | 0.566 ± 0.085 | 0.528 ± 0.087 |
| 592-dim | A | T1 + T2 + T3 | 0.473 ± 0.068 | 0.564 ± 0.068 | 0.563 ± 0.081 |
| 592-dim | D1 | T1 + T2 | 0.595 ± 0.116 | 0.599 ± 0.063 | 0.543 ± 0.083 |
| 592-dim | D2 | T1 + T3 | 0.540 ± 0.119 | 0.575 ± 0.089 | 0.543 ± 0.058 |
| 592-dim | D3 | T2 + T3 | 0.474 ± 0.081 | 0.573 ± 0.051 | 0.516 ± 0.088 |

Note: For the EATD dataset, T1 = Neutral, T2 = Negative, and T3 = Positive.

**Table S13. Summary of statistical significance tests for key comparative claims**

| **Category** | **Comparison Details** | **Statistical Test** | **Effect Size / Mean Difference** | **95% CI** | **p-value** | **q-value (FDR)** | **Significance** | **Description** |
| --- | --- | --- | --- | --- | --- | --- | --- | --- |
| Positive Recall vs. Other Tasks | T4 vs T1 | paired t-test | Cohen's d = 1.64 | [+0.052, +0.110] | <0.001 | <0.001 | *** | Significantly higher than T1 |
|  | T4 vs T2 | paired t-test | Cohen's d = 0.36 | [-0.007, +0.039] | 0.252 | 0.303 | n.s. | Slightly higher than T2, n.s. |
|  | T4 vs T3 | paired t-test | Cohen's d = 0.29 | [-0.008, +0.040] | 0.252 | 0.303 | n.s. | Slightly higher than T3, n.s. |
| 1‑second Speech VS Full‑duration Speech | T1: 1-second vs Full | paired t-test | Cohen's d = 0.23 | [+0.006, +0.021] | 0.002 | **0.003** | ** | Significant |
|  | T2: 1-second vs Full | paired t-test | Cohen's d = 0.36 | [+0.007, +0.027] | 0.003 | **0.003** | ** | Significant |
|  | T3: 1-second vs Full | paired t-test | Cohen's d = 0.18 | [+0.004, +0.019] | 0.006 | **0.006** | ** | Significant |
|  | T4: 1-second vs Full | paired t-test | Cohen's d = 0.74 | [+0.018, +0.036] | <0.001 | **<0.001** | *** | Significant |
| No Synergistic Effect in Multi-task | T4 vs A (T1+T2+T3+T4) | Bootstrap / Permutation Test | Mean diff. = +0.027 | [-0.051, -0.002] | <0.05 | 0.121 | n.s. | No synergy |
|  | T4 vs C1 (T2+T3+T4) | Bootstrap / Permutation Test | Mean diff. = +0.001 | [-0.051, +0.001] | 0.055 | 0.151 | n.s. | No synergy |
|  | T4 vs D6 (T2+T4) | Bootstrap / Permutation Test | Mean diff. = +0.001 | [-0.052, -0.002] | <0.05 | 0.151 | n.s. | No synergy |
|  | Synergy Index (all combinations) | Bootstrap / Permutation Test | — | All crossing zero | > 0.05 | > 0.05 | n.s. | Zero synergy confirmed |
| Window Compensation | Frame-level + Transformer (2s) vs. Statistical + Transformer (10s) | paired t-test | Cohen's d = 0.23 | [-0.121,+0.174] | 0.843 | 0.935 | n.s. | AUC difference n.s.: window shift for trend description |

**Note:** *** q < .001, ** q < .01, * q < .05 (FDR‑corrected); n.s. = not significant.

**Table S14. Twelve acoustic features consistently ranked in top‑20 across all four tasks (with category contribution summary)**

| **Feature Name** | **Acoustic Category** | **T1** | **T2** | **T3** | **T4** |
| --- | --- | --- | --- | --- | --- |
| Spectral_stat_3 | Spectral | 0.02787 | 0.02787 | 0.03105 | 0.02574 |
| Spectral_stat_7 | Spectral | 0.01748 | 0.01897 | 0.01987 | 0.01831 |
| Spectral_stat_2 | Spectral | 0.01578 | 0.01719 | 0.01839 | 0.02069 |
| Spectral_stat_5 | Spectral | 0.01760 | 0.01716 | 0.01713 | 0.01515 |
| Spectral_stat_4 | Spectral | 0.01307 | 0.01614 | 0.01722 | 0.01835 |
| Spectral_stat_0 | Spectral | 0.01240 | 0.01662 | 0.01769 | 0.01723 |
| Spectral_stat_6 | Spectral | 0.01408 | 0.01484 | 0.01690 | 0.01393 |
| Spectral_stat_1 | Spectral | 0.01197 | 0.01360 | 0.01392 | 0.01167 |
| Mel_max_127 | Mel-spectrogram | 0.01381 | 0.01544 | 0.01799 | 0.02015 |
| ZCR_stat_0 | ZCR | 0.01015 | 0.01110 | 0.01110 | 0.00850 |
| ZCR_stat_4 | ZCR | 0.00789 | 0.01120 | 0.00914 | 0.01028 |
| ZCR_stat_2 | ZCR | 0.00671 | 0.01040 | 0.01012 | 0.01098 |
| **Category Contribution Summary** |  | **T1** | **T2** | **T3** | **T4** |
| Spectral (8 features) | — | 26.33% | 26.69% | 27.92% | 26.53% |
| Mel-spectrogram (1 feature) | — | 49.10% | 45.90% | 43.24% | 43.16% |
| ZCR (3 features) | — | 7.05% | 9.39% | 8.64% | 8.34% |
| MFCC (0 in shared set) | — | 9.06% | 11.51% | 12.63% | 12.41% |
| Energy (0 in shared set) | — | 5.81% | 3.19% | 4.38% | 4.79% |
| F0 (0 in shared set) | — | 2.65% | 3.32% | 3.18% | 4.77% |

**Note:** Values in the first 12 rows are mean(|SHAP|). The bottom six rows show the proportion of total SHAP contribution per acoustic category across all features. Bold values in the feature rows indicate the highest SHAP value for each feature across the four tasks. All 12 features consistently rank within the top‑20 most important features across all four tasks.
